# Melatonin-Mediated Circadian Rhythm Signaling Exhibits Bidirectional Regulatory Effects on the State of Hair Follicle Stem Cells

**DOI:** 10.3390/biom15020226

**Published:** 2025-02-04

**Authors:** Yu Zhang, Xuefei Zhao, Shuqi Li, Yanchun Xu, Suying Bai, Wei Zhang

**Affiliations:** 1 College of Wildlife and Protected Area, Northeast Forestry University, Harbin 150040, China; zhangyu2817@nefu.edu.cn (Y.Z.); zhaoxuefei@nefu.edu.cn (X.Z.);; 2 National Forestry and Grassland Administration Research Center of Engineering Technology for Wildlife Conservation and Utilization, Harbin 150040, China; 3 Detecting Center of Wildlife, State Forestry and Grassland Administration, Harbin 150040, China

**Keywords:** melatonin, RORA, *Foxc1*, hair follicle stem cells

## Abstract

The development and regulation of hair are widely influenced by biological rhythm signals. Melatonin plays a crucial role as a messenger in transmitting biological rhythm signals, and its impact on hair development has been well documented. During the process of hair follicle reconstruction, hair follicle stem cells (HFSCs) are the most important cell type, but the regulatory effect of melatonin on the state of HFSCs is still not fully understood. Therefore, it is necessary to conduct a more comprehensive characterization of the effects of melatonin on the state of hair follicle stem cells. The research results indicate that HFSCs express retinoic acid receptor-related orphan receptor alpha (*Rorα*), and melatonin inhibits the expression level of RORA. Experimental results from CUT&Tag, CUT&RUN, and dual luciferase reporter assays demonstrate that *Foxc1* is a downstream target gene of RORA, with RORA regulating *Foxc1* expression by binding to the promoter region of *Foxc1*. The CCK-8 assay results show that low doses of melatonin upregulate the survival rate of hair follicle stem cells, while high doses have the opposite effect. The knockdown of *Foxc1* reverses the inhibitory effect of high-dose melatonin on the survival rate of hair follicle stem cells. Based on these findings, we believe that melatonin-mediated circadian signals exert a bidirectional regulatory effect on the state of HFSCs.

## 1. Introduction

Hair, a unique keratinized skin appendage of mammals, plays a crucial role in the process of environmental adaptation. The hair of mammals is composed of numerous individual hair follicles, and a typical hair follicle includes structures such as the hair bulb, hair shaft, inner root sheath, outer root sheath, sebaceous gland, arrector pili muscle, and connective tissue sheath [1,2]. Hair follicles have specific developmental cycles and are classified into anagen, catagen, and telogen phases based on their morphological characteristics [3,4]. This intrinsic developmental rhythm ensures the lifelong maintenance of coat replacement. At the intersection of the outer root sheath of the hair follicle and the arrector pili muscle, there exists a morphologically protruding area known as the bulge region, where hair follicle stem cells are colonized [5]. There are dozens of cell types involved in the composition of hair follicles, but the prevailing view currently is that hair follicle stem cells are one of the most important driving factors in the process of hair follicle regeneration [6,7,8]. The activation level and correct migration of these cells are crucial for hair follicle development [9,10]. Additional research has demonstrated that HFSCs, besides possessing the common characteristics of adult stem cells, also have a special ability to sense their own state. When HFSCs are overactivated, they can promptly inhibit their own activation level by regulating the expression of the transcription factor FOXC1, thereby preventing premature depletion of the stem cell pool [11]. This is crucial for maintaining the developmental homeostasis of hair follicles. Although HFSCs play a unique role in hair follicle development and cyclical regeneration, the mechanisms by which HFSCs maintain the normal cycle of hair follicles have not been fully elucidated as of yet.

Hair follicle development and cyclical regeneration are influenced by many factors, such as age, hormone levels, gender, and nutritional status [12,13,14,15,16,17]. Among these, circadian rhythm signals are important regulatory factors for hair follicle development [18,19,20]. Melatonin is an amine hormone mainly synthesized and secreted by the pineal gland (vertebrates), which is widely involved in the regulation of physiological processes, especially in the regulation of biological rhythm [21,22,23,24,25]. Light can affect the level of norepinephrine secretion in the superior cervical ganglion through a complex neural signaling system, and this change can further influence the activity of the rate-limiting enzyme for melatonin synthesis in the pineal gland, thereby altering the synthesis and release of melatonin [26,27,28,29,30]. It is due to this inverse relationship between melatonin synthesis and release and light exposure that melatonin serves as a bridge between photoperiod rhythmic signals and biological physiological regulation. Retinoic acid-related orphan receptor alpha (*Rorα*) belongs to the nuclear receptor superfamily and is widely expressed in various cell types throughout the body [31]. It is highly involved in crucial physiological processes such as circadian rhythms, glucose metabolism, and lipid metabolism, and its expression level significantly impacts the state and fate of cells [32,33,34]. Rorα was once thought to be a receptor for melatonin, but new studies indicate otherwise [35]. In addition, there are also studies showing that melatonin is involved in the regulation of *Rorα* expression [36]. However, the regulatory effect of melatonin on *Rorα* in hair follicle stem cells and its potential physiological consequences have not been fully characterized.

Existing research indicates that melatonin has a significant regulatory effect on hair follicle development [37]. Experiments conducted on patients with androgenic alopecia and diffuse alopecia have shown that the topical application of melatonin can significantly increase the proportion of anagen phase hair follicles in the occipital region of patients with androgenic alopecia and in the frontal region of patients with diffuse alopecia [38]. In addition, hair follicles are one of several important extrapineal sites of melatonin synthesis, suggesting that melatonin may also regulate hair follicle development through autocrine and paracrine mechanisms [39]. Other research has also indicated that melatonin can directly influence hair follicle development through the Wnt/β-Catenin signaling pathway [40]. Unfortunately, to date, we still know little about the underlying molecular mechanisms of melatonin in regulating hair follicle development, particularly its role in regulating the state of hair follicle stem cells. Therefore, this study attempts to use hair follicle stem cells as a cellular model and melatonin receptors as an entry point to discuss the impact of melatonin on hair follicle development.

## 2. Materials and Methods

### 2.1. Experimental Design and Drug Treatment

Tissues containing the bulge region were isolated from the whiskers of Sprague–Dawley rats, followed by primary culture of HFSCs using methods from Oshima, Rochat, and Shwartz et al. to establish a cellular model [41,42,43]. We treated the rat HFSCs with melatonin (MCE, HY-B0075) at final concentrations of 500 ng/L, 1000 ng/L (low dose), and 2000 ng/L (high dose) and set up a blank control group. We assessed the effects of different doses of melatonin on the expression of *Rorα* and *Foxc1*, as well as changes in cell viability. Additionally, we analyzed the impact of RORA on *Foxc1* using 10 μM SR1075 (a RORA agonist) and SR3335 (a RORA inhibitor). All animals in this study were treated in accordance with China’s national legislative guidelines on animal welfare. The care and use of experimental animals were approved by the Animal Ethics Committee of Northeast Forestry University.

### 2.2. Immunofluorescence

After the cells had been washed three times with phosphate-buffered saline (PBS), they were fixed with acetone for 15 min. Then, they were permeabilized with 0.5% Triton X-100 at room temperature for 20 min, followed by blocking with goat serum for 30 min at room temperature. The primary antibody was added, and the cells were incubated overnight at 4 °C. After removal of the primary antibody and washing of the cells, the secondary antibody was added, followed by incubation at room temperature for 1 h. After further washing, the coverslip was mounted with anti-fade mounting medium containing DAPI and observed under a fluorescence microscope.

### 2.3. Real-Time qPCR

Total RNA was extracted from the cells and cDNA was synthesized using a Thermo Scientific GeneJET RNA Kit (Thermo Fisher, Waltham, MA, USA, K0732) and PrimeScript™ RT reagent Kit with gDNA Eraser (Takara, Osaka, Japan, RR047A), respectively, following the instructions provided in the kit manuals. Real-time qPCR detection was performed using SsoAdvanced™ Universal SYBR^®^ Green (Bio-Rad, Hercules, CA, USA, 1725270) on a Bio-Rad CFX384 Real-Time PCR Detection System. Relative expression levels were statistically analyzed using the 2^−ΔΔCt^ method. Information regarding all primers used in this study is provided in the Supplementary Materials (Table S1, File S1).

### 2.4. Western Blotting

RIPA buffer containing 1 mmol/L phenylmethanesulfonyl fluoride was used for extraction of total cellular proteins. A BCA Protein Concentration Assay Kit (Solarbio, Beijing, China, PC0020) was used to determine the concentration of each protein sample. The protein loading amount was adjusted to 30 μg. Proteins were thoroughly mixed with 4× protein SDS-PAGE (sodium dodecyl sulfate polyacrylamide gel electrophoresis) loading buffer (Takara, Osaka, Japan, 9173), and the mixture was incubated at 99 °C for 10 min to ensure complete denaturation of the proteins. Depending on the molecular weight of the target protein, 4–12% and 4–20% gels (Genscript, Piscataway Township, NJ, USA, M00653; M00656) were used to separate proteins by SDS-PAGE. Proteins were then transferred onto polyvinylidene fluoride membranes and blocked with 5% bovine serum albumin for 1 h. Then, primary antibodies were added, and the membranes were incubated overnight at 4 °C. Following 1 h incubation of the membranes with HRP-labeled secondary antibodies at room temperature, images were acquired using ECL luminescent solution and a Bio-Rad ChemiDoc MP Imaging System (Bio-Rad, Hercules, CA, USA). Detailed information of the antibody is provided in the Supplementary Materials (Table S2, File S1).

### 2.5. Droplet Digital PCR

EvaGreen Digital PCR Supermix (Bio-Rad, Hercules, CA, USA, #1864034) was used for ddPCR detection. After dilution of the DNA, the reaction system was prepared according to the manufacturer’s instructions and transferred to a droplet generation card. Droplet generation oil (70 μL; Bio-Rad, Hercules, CA, USA, #1864005) was added to each well, and droplets were generated (Bio-Rad, Hercules, CA, USA, #1864002) and transferred to a 96-well plate for PCR reaction. After completion of the reaction, the results were analyzed using a droplet reader (Bio-Rad, Hercules, CA, USA, #1864003) and the Bio-Rad QuantaSoft™ Analysis Pro (QuantaSoft AP) software version 1.4.

### 2.6. Cleavage Under Targets and Tagmentation (CUT&Tag)

CUT&Tag library construction and high-throughput sequencing were conducted by Novogene Bioinformatics Technology Co., Ltd. (Beijing, China). The samples were labeled EG (EG_1, EG_2, EG_3, n = 3), in addition to the IgG control group. The analysis workflow can be outlined in brief as follows: FastQC (v0.11.9) was used for quality control of the raw data to obtain clean reads. Burrows–Wheeler Aligner was used to align the clean reads to the reference genome and analyze the distribution of reads across the genome. The computeMatrix and plotProfile modules of deepTools (v3.2.1) were used to analyze the distribution relative to TSS and relative to the gene body, respectively. MACS2 software (v2.2.9.1) was used for peak detection and statistical analysis of the distribution of peaks across chromosomes. PeakAnnotator (v1.4) and ChIPseeker (v1.40.0) were used for annotation of peaks and statistical analysis of the distribution of peaks across functional regions, respectively. Genes corresponding to the TSS nearest to each peak were considered to be peak-related genes.

### 2.7. Cleavage Under Targets and Release Using Nuclease (CUT&RUN)

To validate the results obtained from CUT&Tag, a CUT&RUN assay was conducted, following the same experimental grouping as used for the CUT&Tag analysis. The CUT&RUN Assay Kit (Cell Signaling Technology, Danvers, MA, USA, #86652) and DNA Purification Buffers and Spin Columns (Cell Signaling Technology, Danvers, MA, USA, #14209) were used to prepare DNA samples from RORA target-binding regions following the manufacturer’s instructions. ddPCR was then used for the detection of the results.

### 2.8. DNA Pull-Down

The DNA pull-down assay was performed using a kit from Sangon Biotech (Shanghai, China, B605112), following the steps described in the instruction manual. Input controls of total cellular proteins and negative controls without probes were set up to ensure the validity of the experiment. After the pull-down was completed, Western blot (WB) analysis was performed on the target protein to determine whether the probe had successfully captured RORA.

### 2.9. RNA Interference

HFSCs with knockdown of the *Foxc1* gene were constructed via transfection with two parallel siRNA targeting the CDS (coding sequence) region of the *Foxc1* gene, using Lipofectamine™ Stem Cell Transfection Reagent (Thermo Fisher, Waltham, MA, USA, STEM00015) and Opti-MEM™ I Reduced Serum Medium (Thermo Fisher, Waltham, MA, USA, 31985070). A negative control group was also set up, consisting of cells transfected with an unrelated siRNA. The steps were performed following the manufacturers’ instructions. After 48 h of transfection of HFSCs, cell viability was detected by CCK-8.

### 2.10. Dual-Luciferase Reporter

The CDS region of the *Rora* gene and the promoter region of the *Foxc1* gene were separately cloned into pCDNA3.1 and pGL3-basic vectors, respectively. After molecular cloning, plasmid extraction was performed. The constructed reporter plasmids were co-transfected with the pRL-TK referenced plasmid into HEK 293T cells. The following groups were set up: pCDNA3.1 and pGL3-basic empty plasmid group; pCDNA3.1 empty plasmid and pGL3-basic-*Foxc1* promoter reporter plasmid group; pCDNA3.1-*Rora* overexpression plasmid and pGL3-basic-*Foxc1* promoter reporter plasmid group; and pCDNA3.1-*Rora* overexpression plasmid, pGL3-basic-*Foxc1* promoter reporter plasmid, and an additional 10μM SR1078 treatment group. Following 48 h of incubation, the Dual-Luciferase^®^ Reporter Assay System (Promega, Madison, WI, USA, E1910) and the FlexStation3 Multi-Mode Microplate Reader (Molecular Devices, San Jose, CA, USA,) were used to detect the results, following the manufacturer’s instructions.

### 2.11. Statistical Analysis

Each experiment included at least three biological replicates, and one-way analysis of variance and Student’s *t*-test were used to assess statistical significance. All statistical analyses were performed using GraphPad Prism 9.5.1 software, and the results were expressed as mean ± standard deviation. *p* < 0.05 was considered to indicate a statistically significant result.

## 3. Results

### 3.1. Characterization and Analysis of Melatonin Receptor Expression in HFSCs

**Figure 1:**
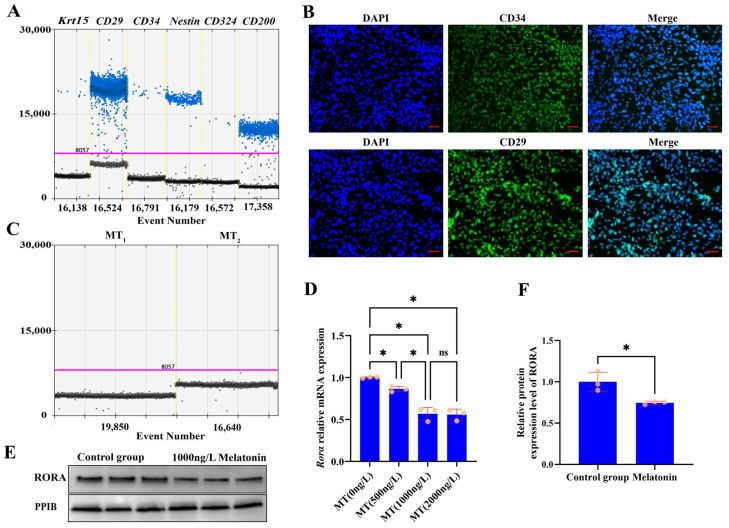
(**A**) Results of ddPCR detection of surface markers of HFSCs in primary cultured cell models; blue dots represent positive signal microdroplets. (**B**) Immunofluorescence staining of CD34 and CD29 (surface markers of HFSCs) in primary cultured cell models; scale bar, 50 μm. (**C**) ddPCR detection of melatonin membrane receptor MT_1_ and MT_2_ expression in HFSCs. (**D**) Effects of different doses of melatonin on the transcriptional level of *Rora*. ns, not statistically significant; * *p* < 0.05. Subsequent significance markers use the same notation. (**E**) WB detection of effects of melatonin (1000 ng/L) on RORA levels. (**F**) Relative expression of RORA in HFSCs after melatonin (1000 ng/L) treatment.

Primary cultured rat hair follicle stem cells are shown in the Supplementary Materials (Figure S1, File S1). Cultured cells were identified based on the reported markers of rat HFSCs [43,44,45,46,47]. The expression of HFSCs markers, including *Krt15*, *CD29*, *CD34*, *Nestin*, *CD324*, and *CD200*, was determined using droplet digital PCR (Figure 1A), and the presence of CD34 and CD29 markers in these cells was confirmed by immunofluorescence (Figure 1B). Having isolated rat HFSCs, we analyzed the expression of melatonin receptors in these cells using ddPCR. No expression of melatonin membrane receptors MT1 and MT2 was detected in primary rat HFSCs (Figure 1C). To investigate the effects of melatonin on *Rorα* levels in HFSCs, we treated the cells with melatonin at concentrations of 500 ng/L, 1000 ng/L, and 2000 ng/L for 48 h. Compared with the control group, melatonin treatment at any dose reduced the transcriptional level of *Rorα*; a dose-dependent effect was observed as the melatonin dose was increased, but there was no statistically significant difference between the effects of 1000 ng/L and 2000 ng/L melatonin treatments (*p* > 0.05, Figure 1D). The Western blotting results also confirmed that treatment with 1000 ng/L melatonin suppressed the levels of RORA in HFSCs (Figure 1E,F).

### 3.2. CUT&Tag

**Figure 2:**
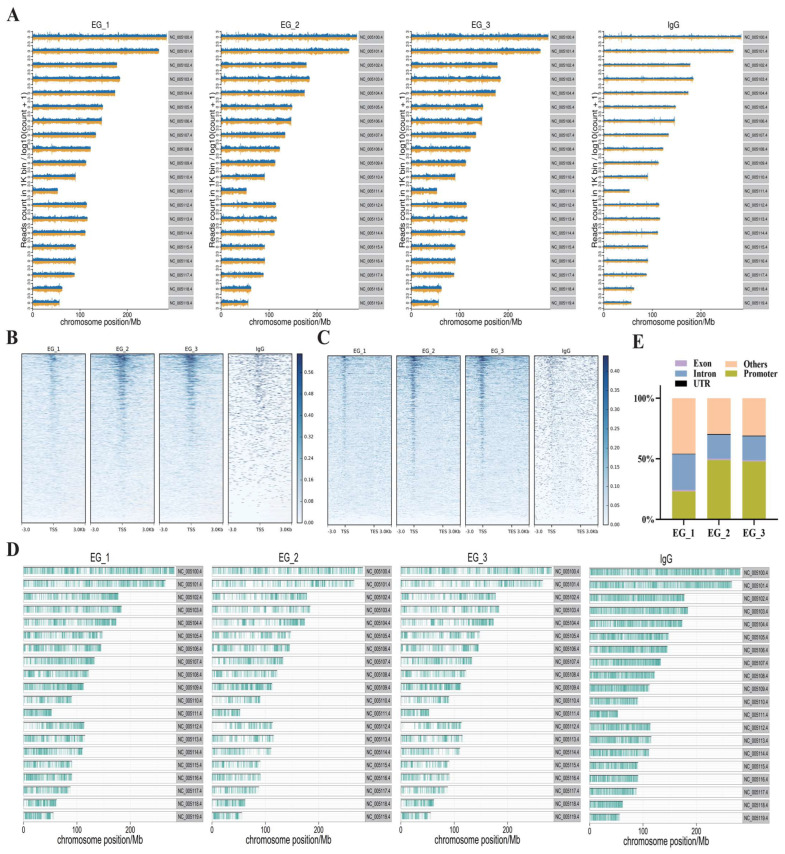
(**A**) Distribution of reads on chromosomes (experimental group vs. IgG control group). (**B**) Distribution of reads within 3 kb upstream and downstream of transcription start sites. (**C**) Distribution of reads across the gene body, with a higher concentration of reads located in the region of transcription start sites. (**D**) The distribution of peak signals at RORA binding sites along the chromosome. (**E**) The statistical results of peak signals at RORA binding sites located at various positions within genes.

**Figure 3:**
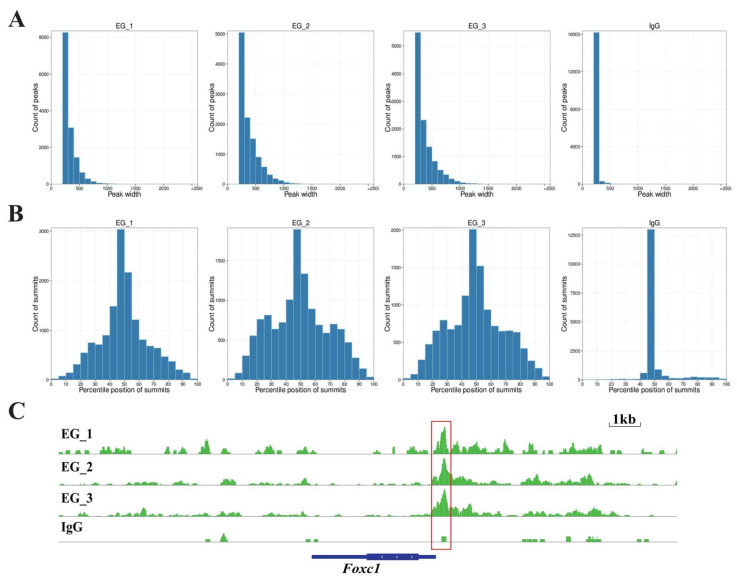
(**A**) The statistical results of the length range of peak signals at RORA binding sites. (**B**) The statistical results of the relative position of peak summits indicate that most of the summits are located in the middle of the peak signals. (**C**) The peak signal of the RORA binding site present in the promoter region of the *Foxc1* gene, which is indicated by the red box in the diagram.

To determine the effects of RORA on hair follicle stem cells, we utilized CUT&Tag (Cleavage Under Targets and Tagmentation) technology to screen for downstream target genes directly regulated by RORA. We also set up an IgG control group to minimize the effects on the results of interference by background signals. First, we analyzed the distribution of reads on the chromosome by setting a 1 kb sliding window (Figure 2A). Subsequently, we statistically analyzed the distribution of reads within 3 kb upstream and downstream of the transcription start sites (TSSs) and found that the read signal intensity was highest near the TSS (Figure 2B). In addition, we examined the distribution of reads relative to the gene body and found that the read signal intensity at the TSS was significantly higher than at other regions such as the transcription end site (Figure 2C). After peak calling using MACS2 software, we analyzed the distribution of peaks on the chromosome and counted the proportions of peak distribution positions (Figure 2D,E). Most peaks had a width of less than 500 bp, with the peak summit located in the middle of the peak (Figure 3A,B). It is noteworthy that we observed a significant peak signal in the promoter region of *Foxc1* in the CUT&Tag results, suggesting that *Foxc1* may be a direct downstream target gene of RORA (Figure 3C). Considering previous research findings regarding the regulatory role of the *Foxc1* in the state of hair follicle stem cells, we focused our attention on the binding relationship between RORA and the *Foxc1* promoter region, as well as their potential regulatory effects.

### 3.3. Melatonin May Regulate Foxc1 Expression Through RORA to Prevent Excessive Activation of HFSCs

**Figure 4:**
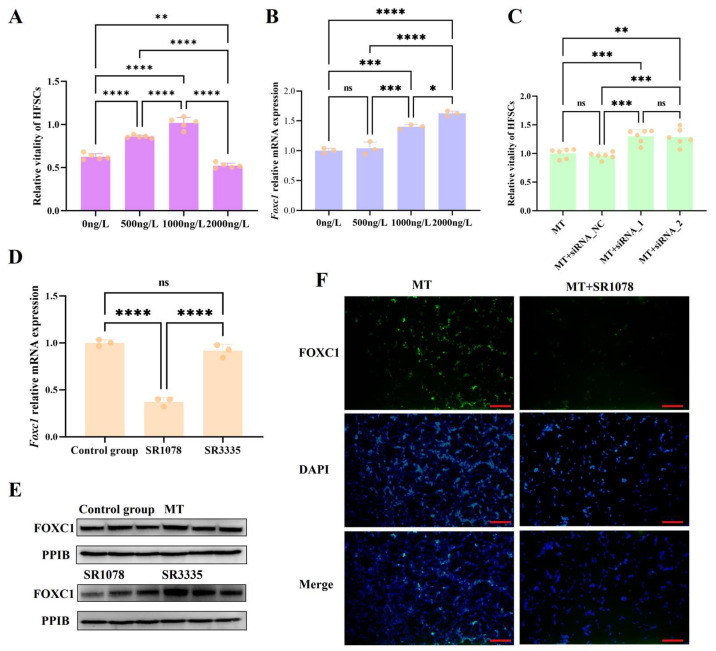
(**A**) Changes in the viability of hair follicle stem cells under treatment with different doses of melatonin; ** 0.001 < *p* < 0.01, **** *p* < 0.0001. Subsequent significance markers use the same notation. (**B**) Changes in the mRNA levels of the *Foxc1* gene in hair follicle stem cells under treatment with different doses of melatonin; ns means 0.05 < *p*, * 0.01 < *p* < 0.05, *** 0.0001 < *p* < 0.001. Subsequent significance markers use the same notation. (**C**) Knockdown of the *Foxc1* gene partially alleviates the inhibitory effect exerted by high-dose melatonin (2000 ng/L) on the viability of hair follicle stem cells. (**D**) The qPCR detection results for the relative transcription level of *Foxc1* gene (the final concentrations of SR1078 and SR3335 are 10 μM). (**E**) Treatment of hair follicle stem cells with the RORA agonist SR1078 and antagonist SR3335 affected the expression levels of the FOXC1 protein (the final concentration of melatonin is 2000 ng/L; the final concentration for SR1078 and SR3335 is 10 μM). (**F**) Immunofluorescence staining results for the FOXC1; scale bar, 100 μm (the final concentrations of melatonin and SR1078 are 2000 ng/L and 10 μM, respectively).

We treated HFSCs with different doses of melatonin and found that low doses of melatonin (0 ng/L, 500 ng/L, and 1000 ng/L) significantly upregulated the viability of hair follicle stem cells, whereas high doses (2000 ng/L) exhibited an inhibitory effect on their viability (Figure 4A). Then, we treated HFSCs with various doses of melatonin and measured the expression levels of the *Foxc1* gene. As the dose of melatonin increased, the mRNA level of *Foxc1* gradually rose (Figure 4B). This indicates a transition of HFSCs from an activated state to a quiescent state, suggesting that the upregulation of *Foxc1* by high levels of melatonin is a crucial factor that prevents the overactivation of HFSCs. Furthermore, the knockdown of *Foxc1* expression by RNA interference enhanced the viability of HFSCs treated with high doses of melatonin (Figure 4C). In addition, the FOXC1 expression was significantly downregulated after RORA activation (Figure 4D,F). The above results indicate that the impact of melatonin and its receptor-dependent rhythm signals on the state of HFSCs depends on the signal intensity. Fluctuating rhythm signals can exert different effects on HFSCs, with RORA and FOXC1 playing crucial roles in this process. Melatonin regulates the level of *Foxc1* through RORA to prevent the overactivation of HFSCs, which is also a crucial aspect of maintaining their homeostasis.

### 3.4. Verification of Interactions Between RORA and Foxc1

**Figure 5:**
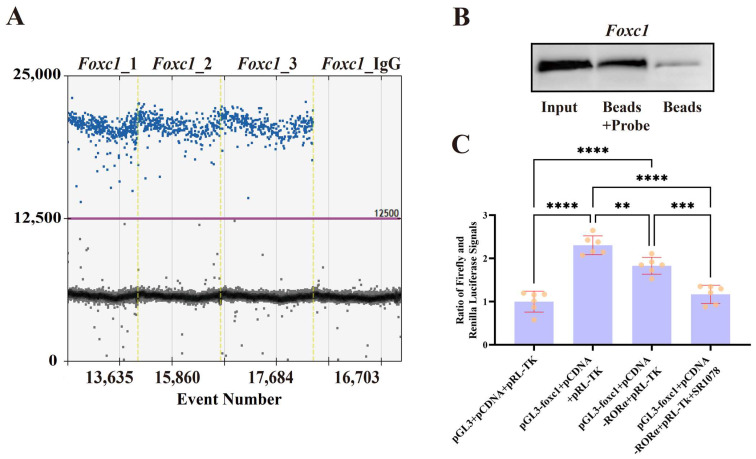
(**A**) The ddPCR detection results of the CUT&RUN-enriched DNA samples showed that the experimental group was positive while the control group was negative, indicating that RORA can bind to the *Foxc1* promoter region. (**B**) The results of the DNA pull-down experiment demonstrate that the probe targeting the *Foxc1* promoter region has the ability to bind to the RORA protein. (**C**) The results of the dual-luciferase reporter assay indicate that RORA can bind to the promoter region of *Foxc1* and downregulate the transcription level of *Foxc1*. ** 0.001 < *p* < 0.01, *** 0.0001 < *p* < 0.001, **** *p* < 0.0001.

The analysis of the CUT&Tag results indicated that the expression of *Foxc1* may be directly regulated by RORA. To validate the interactions of RORA and *Foxc1*, we used CUT&RUN to enrich DNA fragments potentially bound by RORA in the genome, as well as setting up an IgG control. We then used ddPCR to detect the potential binding sites of RORA in the promoter regions of *Foxc1*. There were positive results for *Foxc1*, whereas the IgG control group showed negative results, indicating that RORA interacts with these genes under physiological conditions (Figure 5A). Given that *Foxc1* occupies a unique position in regulating the state of HFSCs, we used biotin-labeled promoter region probes to analyze the binding relationship of RORA with the promoter regions of the *Foxc1* genes by DNA pull-down and WB assays; the probes successfully captured the RORA protein, compared with the probe-free control group, indicating that RORA has the ability to bind to the promoter region of the *Foxc1* genes in vitro (Figure 5B). To further confirm, we used dual-luciferase reporter assays to validate the binding relationship of RORA with the *Foxc1* promoter region and the transcriptional regulatory effects resulting from this binding (Figure 5C). The results indicated that RORA negatively regulates *Foxc1*, consistent with the findings of previous studies. In addition, given the potential impact of a lack of RORA ligands in vitro on the transcriptional regulation of *Foxc1*, we performed an additional treatment with SR1078. The results showed that SR1078 further enhanced the negative regulatory effect of RORA on *Foxc1*.

## 4. Discussion

Melatonin exerts its physiological effects largely through its receptors. However, the findings of this study indicate that membrane receptors for melatonin are not expressed in rat hair follicle stem cells. This suggests that the recently discovered potential nuclear receptors for melatonin, *AhR* and *PPARγ*, may play a significant role in the regulation of HFSCs by melatonin [48]. Extensive evidence indicates that melatonin and its receptor-dependent rhythmic signal transduction play crucial regulatory roles in hair follicle development. In particular, the melatonin-synthesizing capacity of skin tissue and the relatively short half-life of melatonin may be important in the regulation of hair follicle development [49]. However, the underlying molecular mechanisms responsible for this phenomenon remain elusive. HFSCs play a pivotal role in the cyclic reconstruction of hair follicles. Preliminary studies have explored the potential relationship between melatonin and HFSCs, but a more thorough evaluation of their interaction is still necessary [40,50,51]. In this study, we isolated hair follicle stem cells from rats for use in our experimental model and examined the expression of melatonin receptors in these cells. Although previous studies have shown that the physiological effects of melatonin largely depend on its receptors, we surprisingly found that our cellular model did not express membrane melatonin receptors.

After treating hair follicle stem cells with different doses of melatonin, we found that melatonin inhibits the expression of *Rorα*. To further analyze the physiological significance of melatonin-induced inhibition of *Rorα* expression in regulating the state of HFSCs, we identified potential downstream target genes of RORA using CUT&Tag. The exciting discovery was the peak signal in the *Foxc1* promoter region, suggesting that this key transcription factor, which regulates the viability of HFSCs, may be a direct downstream target gene of RORA. To further validate this hypothesis, we first used CUT&RUN in combination with ddPCR to detect the presence of *Foxc1* promoter fragments in the enriched RORA-binding segments. Subsequently, we employed DNA pull-down technology to reversely verify their binding relationship. Additionally, we conducted dual-luciferase reporter experiments to demonstrate the regulatory relationship between the two, and the results are consistent with those obtained from qPCR, WB, and IF. Thus, we have proven that RORA downregulates the expression level of *Foxc1* by directly binding to its promoter region, and melatonin regulates the expression of *Foxc1* by downregulating the expression of RORA.

The role of the *Foxc1* gene in regulating the state of hair follicle stem cells and in hair follicle development has gradually been unveiled in recent years. Hair follicle stem cells need to maintain their stem cell reservoir by minimizing cell division during the catagen and telogen phases of the hair cycle while rapidly entering an activated state during the anagen phase to provide the cellular basis for hair follicle reconstruction. This periodic state switching is essential for proper hair follicle development. In this periodic state switching process, *Foxc1* plays a crucial role. Studies have shown that when *Foxc1* is knocked out, hair follicle stem cells in the active phase cannot re-establish a resting state, leading to the premature depletion of hair follicle stem cells. This may be achieved through the activation of Nfatc1 and bone morphogenetic protein signaling pathways by *Foxc1* [11]. In this study, we found that low-dose melatonin treatment significantly enhanced cell viability, which is not surprising considering our previous understanding of melatonin. However, high-dose melatonin treatment resulted in decreased cell viability. This indicates that melatonin has different dose effects on regulating the viability of hair follicle stem cells. When we knocked down the *Foxc1* gene, the inhibitory effect of melatonin on hair follicle stem cell viability diminished, suggesting that the upregulation of *Foxc1* levels by high-dose melatonin may be one of the important reasons for its inhibitory effect on hair follicle stem cell viability. The inhibition of RORA expression by melatonin and the regulatory relationship between RORA and *Foxc1* are among the important molecular mechanisms underlying this process. *Foxc1* may act as a brake gene in the upregulation of hair follicle stem cell vitality through melatonin-mediated circadian signaling, preventing its own excessive consumption.

The results of this study indicate that melatonin-dependent endogenous rhythmic signals have a bidirectional regulatory effect on the state of hair follicle stem cells. Within a certain threshold, these signals enhance the viability of hair follicle stem cells, promote the cell cycle, and complete hair follicle reconstruction. Exceeding this threshold, however, results in the inhibition of cell viability, helping to maintain the stem cell pool and thereby avoiding the premature depletion of hair follicle stem cells. This intrinsic biological rhythm signaling pathway has the potential to become a therapeutic target for treating issues such as alopecia. Additionally, the *Foxc1* gene, as a downstream target gene of RORA, participates in regulating the developmental state of hair follicle stem cells. However, its underlying molecular mechanisms have not been fully characterized. In-depth exploration of the pathways through which *Foxc1* regulates the state of HFSCs in future research is necessary, as it will help provide a theoretical basis for further elucidating the physiological mechanisms of hair follicle development.

## 5. Conclusions

In conclusion, melatonin downregulates the expression level of the *Rorα* gene in rat hair follicle stem cells. Low doses of melatonin upregulate the viability of HFSCs, while high doses have the opposite effect. Upon activation, RORA regulates the transcription level of the *Foxc1* gene by binding to its promoter region, and alterations in *Foxc1* gene expression play a crucial role in the process of melatonin regulating HFSCs viability.

## Data Availability

The original contributions presented in this study are included in the article/Supplementary Material. Further inquiries can be directed to the corresponding authors.

## Supplementary Materials

The following supporting information can be downloaded at: https://www.mdpi.com/article/10.3390/biom15020226/s1, Figure S1. Primary culture of hair follicle stem cells in rats; Table S1: The primers used in this research; Table S2: The primary antibody used in this research; Table S3: Data Source; File S1: Original images for western blots.
