# Supplementary material for: Melatonin-Mediated Circadian Rhythm Signaling Exhibits Bidirectional Regulatory Effects on the State of Hair Follicle Stem Cells
Source: Biomolecules. 2025 Feb 4;15(2):226. doi: 10.3390/biom15020226 (PMC11852975; doi:10.3390/biom15020226)
Supplement: Supplementary file 1 [file biomolecules-15-00226-s001.zip › Supplementary Materials.pdf]

Supplementary materials for “Melatonin-mediated circadian rhythm signaling exhibit bidi-rectional regulatory effects on the state of hair follicle stem cells”

Yu Zhang *et al.*

*Corresponding author. Email: zhangyunefu@163.com

This file includes:

Figure S1.
Tables S1 to S2.

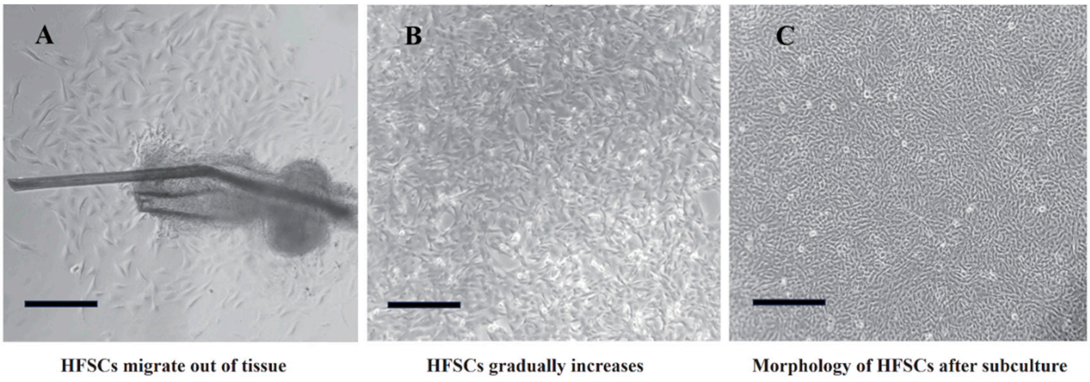

Figure S1. Primary culture of hair follicle stem cells in rats. (A) After 4 days of primary culture, cells can be observed migrating from the bulge region. Scale bar is 250μm. (B) With further expansion culture, the cell density gradually increases, and the HFSCs cultured in vitro exhibit good proliferative vitality. Scale bar is 100μm. (C) The morphology of high-density HFSCs after subculture. Scale bar is 100μm.

Table S1. The primers used in this research

Primer Name	Sequence 5'-3'
CD200F	TCAGCCCACCTACAGAGACA
CD200R	GAGTAAGGCAAGCTGTCCCA
CD324F	CGTACCAGGGTCAAGTGCTT
CD324R	CAGAATGCCCTCGTTGGTCT
CD29F	CTCCTTCCCTGAGTGGCAAG
CD29R	GTGTCTCAGGGAACATGGCA
Krt15F	TTGTGGTTTTGGTGGAGGCT
Krt15R	AGCTCTGTGTTGGCCTCTTC
NestinF	AGCACTCCCATCCCACCTAT
NestinR	GGGTTGTGGCTAAGGAGGTC
CD34F	CGTACCAGGGTCAAGTGCTT
CD34R	CAGAATGCCCTCGTTGGTCT
PPIBF	GGCACAGGAGGAAAGAGCAT
PPIBR	ACCACATCCATGCCTTCCAG
RORAF	AGGCTCGCTAGAGGTGGTGTT
RORAR	CGTCTTCGGTCAGGTGCATAGA
Foxc1F	GCAGCCCAAGGACATGGTGAAG
Foxc1R	GCAGCCCAAGGACATGGTGAAG
Foxc1 for CUT F	AGTGGGTGGGTGCGGTAGTA
Foxc1 for CUT R	GAGACGCCTGTGAATGAGCCA

Table S2. The primary antibody used in this research

Protein	Information
CD34	Abcam ab81289
ITGB1	Proteintech Group 12594-1-AP
RORα (for WB, CUT&Tag and CUT&RUN)	Abcam ab256799
RORα (for DNA pull-down)	Santa Cruz sc-518081
PPIB	Proteintech Group 11607-1-AP
FOXC1	Affinity #DF3252
